# Molecular profiling of epigenetic landscape of cancer cells during extracellular matrix detachment

**DOI:** 10.1038/s41598-021-82431-w

**Published:** 2021-02-02

**Authors:** Mohammad Imran Khan, Mazin A. Zamzami, Aftab Ahmad, Hani Choudhry

**Affiliations:** 1 Department of Biochemistry, Faculty of Science, King Abdulaziz University, Jeddah, 21589 Saudi Arabia; 2 Cancer Metabolism and Epigenetic Unit, Faculty of Science, King Abdulaziz University, Jeddah, 21589 Saudi Arabia; 3 Department of Health Information Technology, Faculty of Applied Studies, King Abdulaziz University, Jeddah, Saudi Arabia

**Keywords:** Cancer microenvironment, DNA methylation

## Abstract

During cancer, a major challenge faced by oncologists is the treatment of metastasis; a leading cause of cancer-related deaths around the world. Metastasis involves a highly ordered sequence of events starting with the detachment of tumor cells from the extracellular matrix (E.C.M.). In normal cells, detachment from E.C.M. triggers programmed cell death, termed anoikis. However, tumor cells dodge their way to anoikis and spread to distant sites for initiating the metastatic program. In this work, we explored the impact of E.C.M. detachment on the expression of some major oncogenic histone methyltransferases. Results showed both EZH2 expression and its enzymatic activity were significantly increased in E.C.M. detached cancer cells when compared to the attached cells. Inhibition of EZH2 results in a significant reduction in cell proliferation, spheroids size, and induction in apoptosis in E.C.M. detached cells. Furthermore, we observed a reduction in EZH2 expression levels in single cells when compared to clusters of E.C.M. detached cells. Finally, we combined the EZH2 inhibition with AMPK, known to be highly expressed in E.C.M. detached cancer cells and observed antagonistic effects between the two pathways. The observed results clearly showed that E.C.M. detached cancer cells require oncogenic EZH2 and can be targeted by EZH2 inhibitors.

## Introduction

Cancer, a fatal disease, challenges human health to become the major contributor to deaths among diseased populations around the world. In the recent past, we have witnessed a great deal of advancement in diagnosis, prognosis, and treatment options for patients. However, despite that, a large number of patients die mainly because of metastasis of primary tumors (History of cancer A.C.S.)^1^. During metastasis, a cancer cell manages to escape from its primary site and try to survive using perfidious relocation and navigation via the lymphovascular system and ultimately form tumors in secondary organs. Recent work had clearly pointed towards the tumor-promoting role of the tumor microenvironment, an important factor of cancer biology additionally to the well-defined genetic components of cancer development^2–6^. The key features of the tumor microenvironment of a solid tumor include hypoxia, E.C.M. (extracellular matrix), and multiple types of cells, all of which advance as the tumor progresses in growth.

Anoikis, a specialized cell death program, primarily occurs during the loss of contact of epithelial cells with the cell-extracellular matrix (E.C.M.). This highly regulated program begins at the cell’s surface mainly via anoikis initiating keys molecules, namely death receptors (D.R.s), cadherins, and integrins^7^. At the start of the anoikis program, major morphological changes i.e. activation, conformational changes, and dimerization of ligands of the cell surface lead towards the start of a series of molecular events mainly leading towards cell death^8^. This cell death can be either mitochondrial-mediated apoptosis signaling (intrinsic) or caspase-8-mediated apoptosis signaling (extrinsic). One such example is the activation of integrin pathways, formation of integrin oligomer, which ultimately leads to the activation of the intrinsic apoptosis signaling in epithelial cells during anoikis. Similarly, loss of cellular contact with the E.C.M. induces binding of death receptors with their cognate ligands in the proximity, leading to extrinsic apoptosis signaling^9^.

In the course of the metastatic pathway, for cells to develop resistance to anoikis, they must possess a survival strategy that involves avoiding attaching themselves to nearby cells and also to the original ECM^10^. Metastatic cells, therefore, must bypass anoikis under two conditions. (1): a condition in which it enters the bloodstream after the tumor cells detached themselves from the primary tumor and hence the E.C.M. (2): a condition that results in different signaling from the primary tumor microenvironment due to its arrival at a secondary having a differential E.C.M. composition. Anoikis is important in inhibiting oncogenesis, particularly metastasis, mainly by targeting and eliminating primary tumor cells that have lost E.C.M. attachment^11^. According to Yu et al.^12^, cells undergo apoptosis after losing contact between the malignant cells and the nearby tumor cells and their E.C.M. Nonetheless, the metastatic pathway is a well-ordered process where primary tumor cells detach from the primary site and invade the lymphatic system, facilitating the traveling of these primary tumor cells mainly to adhere and develop tumors at secondary sites^13^. Since there is a difference in the E.C.M. composition between the secondary and the primary tumor sites and following the last step of metastasis, these metastasis cells must therefore bypass anoikis.

Currently, with growing information, cancer is defined as both genetic and epigenetic disease^14^. Genetic alterations are alone not enough to understand and explain the complex nature of cancer cells^15^. Understanding the epigenome offers an additional elucidation of reasons and mechanisms, along with genetics, of the metastatic process during cancer. The contribution of epigenetic alterations is not only limited to the early phase of transformation or initiation but also it is now widely accepted that it plays a significant role in the metastasis of epithelial cancers. Further, the process of epithelial-mesenchymal-transition that plays an essential part in the complex process of metastasis is also epigenetically regulated^16^. Despite the pieces of evidence, a clear understanding of the epigenetic landscape during E.C.M. detachment in cancer cells is lacking. Therefore in the current work, we explored the expression of major metastasis-associated histone modifiers like EZH2, EHMT1/2, ARID1D, DOT1L, and CARM1^17–22^ and probed their potential role in the survival of different epithelial cancers cell lines during E.C.M. detached conditions. This information will be vital for designing new therapy regimes (adjuvant or combination therapies) using epigenetic drugs along with the conventional chemotherapies for cancer patients.

## Results

### EZH2, a histone methyltransferase, is highly expressed in matrix detached conditions

**Figure 1:**
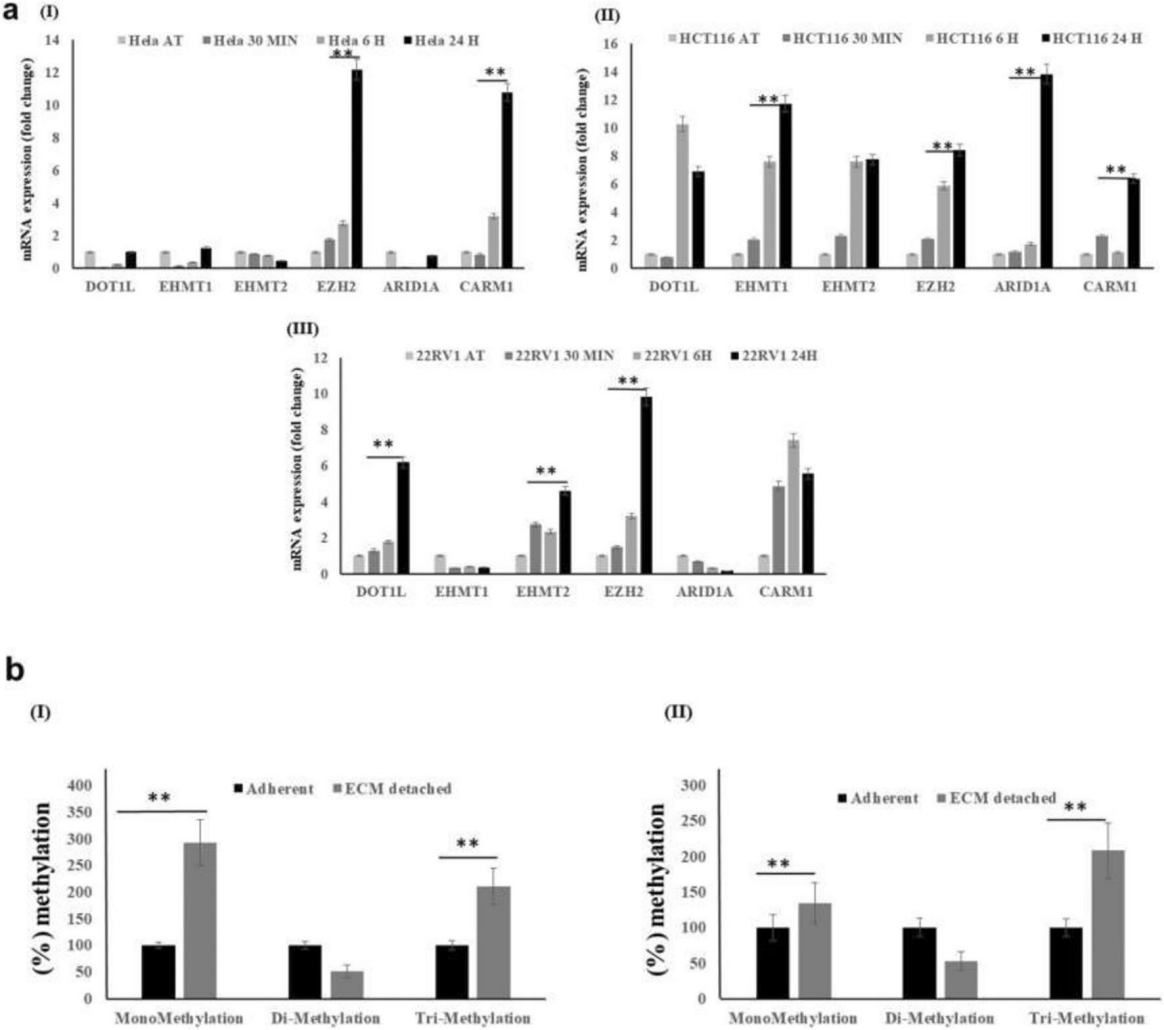
E.C.M. detachment induces EZH2 methyltransferase expression and activity. (**a**) All three endogenous cancer cells were grown in an ultra-low attachment plate for 30 min, 6 h, and 24-h. After a specific time point, R.N.A. was isolated, and quantitative-PCR was performed for targeted genes, values were normalized with housekeeping gene RPLP0. Error bars illustrate mean ± S.E.M. (**b**) H3K27 mono, di, and trimethylation activity assay of the nuclear lysates of HCT116 and HeLa during E.C.M. detached vs. attached condition.

In order to understand the global impact of E.C.M. detachment on cancer cells' methylome, we collected the R.N.A. from three different cell lines (HeLa, HCT116, and 22Rυ1) cultured in both attached and E.C.M. detached conditions. We primarily focused on exploring the expression of histone methyltransferases, namely EZH2, EHMT1, EHMT2, DOT1L, ARID1A, and CARM1, that are well associated with primary tumorigenesis and distant metastasis in a wide variety of cancers^17–22^. Data showed a statistically significant induction in the expression of EZH2, and CARM1 methyltransferases in E.C.M. detached when compared with the attached HeLa cell line (Fig. 1aI). Further, we observed a statistically significant induction in the expression of EZH2, CARM1, and DOT1L methyltransferases in E.C.M. detached when compared with the attached 22Rυ1 cell line (Fig. 1aII). Finally, we also noticed a strong and statistically significant induction in the expression of EZH2, CARM1, DOT1L, EHMT1, and ARID1A methyltransferases in E.C.M. detached when compared with the attached HCT116 cell line (Fig. 1aIII). Since EZH2 was the most consistent upregulate methyltransferase among all in all the three cell lines tested, we, therefore, selected EZH2 methyltransferase for further analysis. In addition, its role in both primary and metastatic tumors are well studied. However, information regarding its expression and function in cancer cells during E.C.M. detachment is not established yet.

Next, we performed quantitative analysis for H3K27 mono/di/trimethylation (H3K27me1/2/3) in nuclear lysates, a product of EZH2 methyltransferase enzymatic activity in E.C.M. detached cancer cell lines, and compared it with attached conditions. As shown in Fig. 1b, we clearly observed a statistically significant induction in H3K27me1 levels in both HCT116, and 22Rυ1 cell lines in E.C.M. detached conditions when compared to attached conditions, respectively. We also observed a clear trend of increase in H3K27me3 levels (not statistically significant) in both cell lines during the E.C.M. detached conditions when compared to attached conditions respectively. Additionally, we measured the expression of H3K27 demethylases, namely KDM6A and KDM6B. We found an increased expression of both KDM6A and B in HeLa and 22Rυ1 cell lines. In contrast, in HCT116 cell lines, only KDM6A was significantly induced in E.C.M. detached conditions when compared to attached (Supplementary Fig. S1). Overall, the above data confirm the increased expression and activity of EZH2 methyltransferase activity in E.C.M. detached conditions.

### Targeting histone methyltransferase EZH2 reduces proliferation and spheroid size in matrix detached conditions

**Figure 2:**
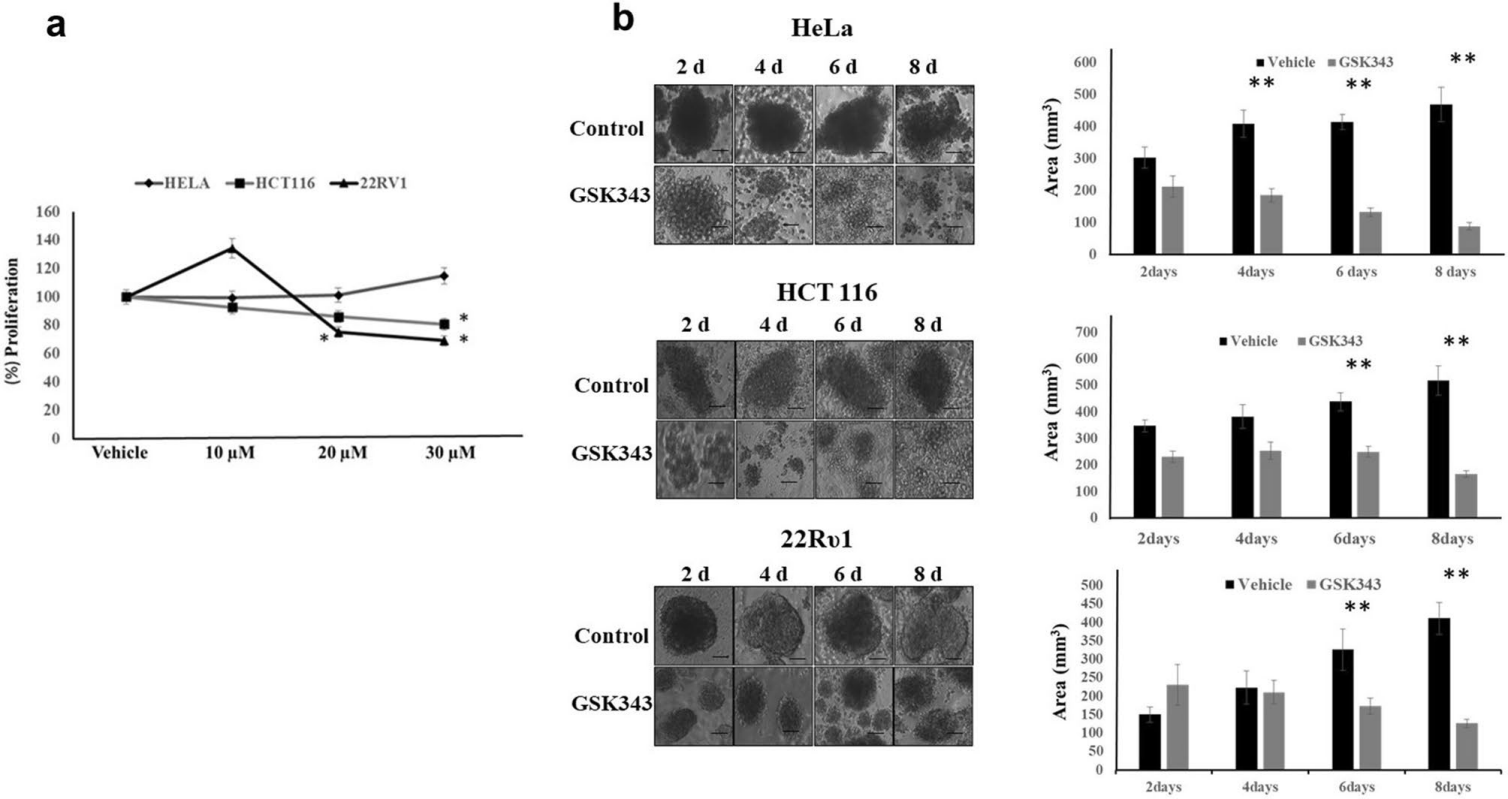
EZH2 methyltransferase activity inhibition by GSK343 reduces cell viability and sphere-forming capability. (**a**) Different cancer cell lines, namely 22Rυ1, HCT116, and HeLa, were treated with GSK343 and EZH2 specific inhibitor, and cell viability assay was performed at 48 h; *P < 0.05. (**b**) Cancer cells HeLa, HCT116, and 22Rυ1 were grown in low attachment plates, and E.C.M. detached cells (spheres) were treated with different concentrations of GSK343 for 2–8 days, and images were captured by Nikon inverted light microscope at ×40. Images were analyzed for size measurement using image J software (mean ± S.E.M., n = 8). *P < 0.05. A total number of 100 spheroids were quantified for size analysis.

In order to confirm EZH2 as a therapeutic target for the E.C.M. detached cancer cells, we decided to use an EZH2 methyltransferase activity inhibitor, namely GSK343, which is known to efficiently inhibit the activity of EZH2. Treatment of E.C.M. detached cancer cell lines with GSK 343 results in a statistically significant reduction in cell proliferation of HCT116 and 22Rυ1 cell lines (20% in HCT116 and 40% in 22Rυ1 approximately during GSK343 treatment when compared with untreated). We fail to see any significant reduction in proliferation in E.C.M. detached HeLa cell line when compared to the attached (Fig. 2b). In addition, we performed the cell proliferation assay in the attached condition using the above-mentioned cell lines and found low sensitivity of GSK 343 for inhibition of cell proliferation in the attached condition when compared with E.C.M. detached (Supplementary Fig. S2).

Since E.C.M. detachment of cancer cells results in the formation of spheroids, we assess the impact of GSK 343 treatment on spheroid size. As shown in Fig. 2b, E.C.M. detachment of HeLa cell lines results in a statistically significant and gradual increase in spheroid size with an increase in time, i.e., from 303 mm^3^ on the 2nd day to 469 mm^3^ on the 8th day. Treatment of GSK343 dramatically reduces the spheroid size on the 8th day, i.e., from 469 to 88 mm^3^. Further, we observed a similar increase in spheroid size in E.C.M. detached HCT116 cells i.e., from 318 mm^3^ on the 2nd day to 548 mm^3^ on the 8th day and GSK 343 treatment significantly reduces the spheroid size from 548 to 166 mm^3^ on the 8th day (Fig. 2b). Next, in E.C.M. detached 22Rυ1 cells, we observed a similar increase in spheroid size, i.e., from 150 mm^3^ on the 2nd day to 411 mm^3^ on the 8th day and like the previous results with other cell lines, GSK343 treatment significantly reduces the spheroid size from 411 to 126 mm^3^ (Fig. 2b). Overall, the above data clearly showed that targeting EZH2 with a specific inhibitor (GSK343) not only reduces proliferation but also attenuates spheroid formation in E.C.M. detached cancer cells.

### Cell clustering is important for high EZH2 expression during matrix detached condition of cancer cell lines

**Figure 3:**
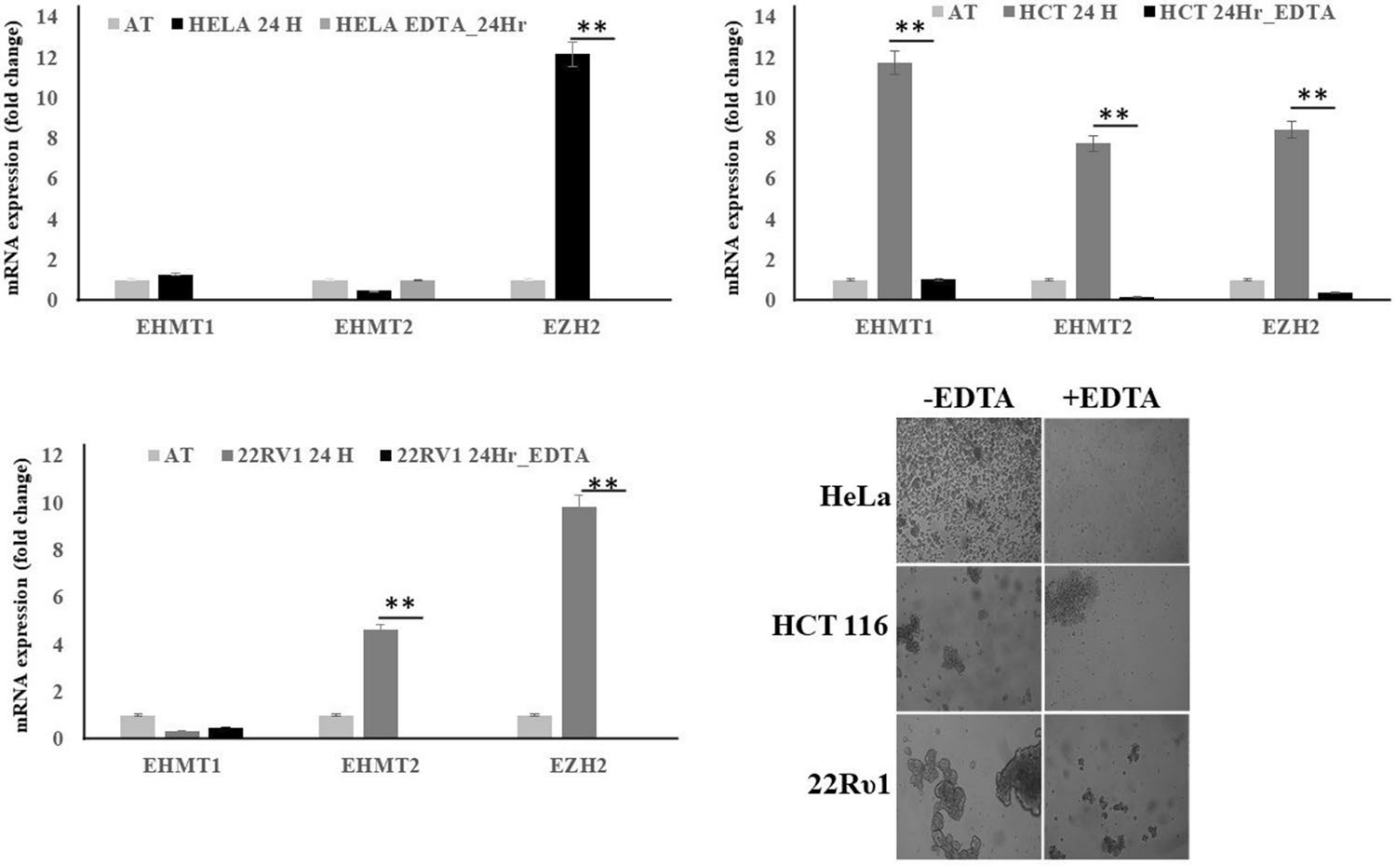
Cell clustering is critical for EZH2 induction during matrix detachment. All three endogenous cancer cells were grown in ultra-low attachment plates both in the presence and absence of EDTA for 24-h. Furthermore, after completion of the time point, R.N.A. was isolated, and quantitative-PCR was performed for targeted genes, values were normalized with housekeeping gene RPLP0. Bars depicting normalized data (mean ± S.E.M., n = 3), *P < 0.05.

Recent studies have shown that E.C.M. detached cancer cells essentially need to form clusters in order to do a metabolic switch from oxidative phosphorylation to glycolysis to maintain cell proliferation and vitality^17^. Since inhibition of EZH2 was reducing the cell proliferation, we were interested in finding out whether clustering was important for high EZH2 expression in E.C.M. detached cancer cells. In order to assess this, we detached various cell lines from E.C.M. and forced them to grow in the presence of EDTA, which is an inhibitor of clustering. Cells in the presence of EDTA fail to induce the expression of EZH2 methyltransferase (Fig. 3), clearly suggesting that clustering was essential for an increase in EZH2 methyltransferase levels like other critical factors i.e. hypoxia and glycolytic switch for the survival of E.C.M. detached cancer cell lines. These results also suggest that targeting non-clustered (single) E.C.M. detached cells with EZH2 methyltransferase specific inhibitors like GSK343 will not benefit as much as it will benefit the clustered (spheroids) E.C.M. detached cells.

### Dual AMPK and EZH2 inhibition induce spheroid size reduction and apoptosis capability in matrix detached conditions

**Figure 4:**
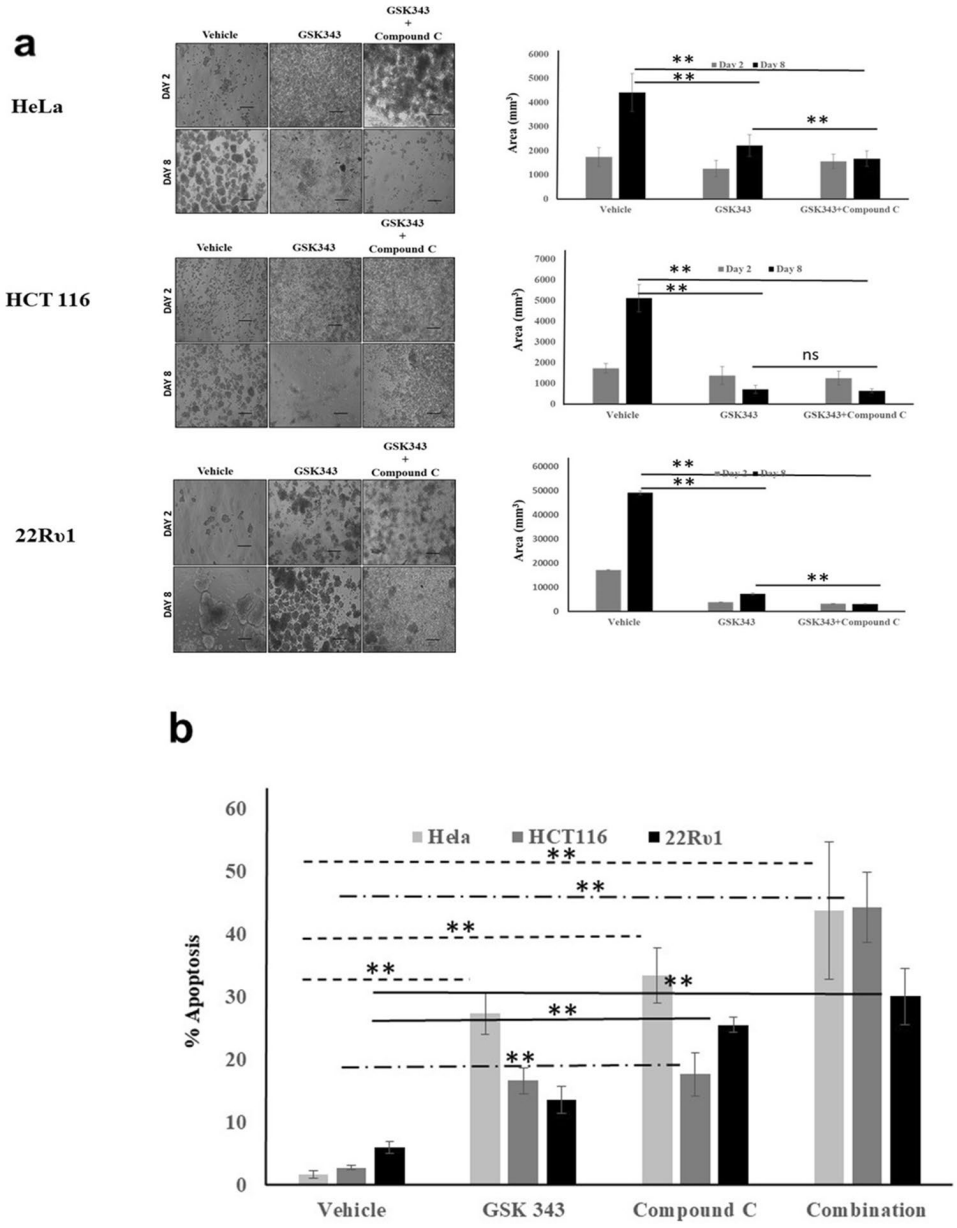
A combination of EZH2 and AMPK inhibitor synergize to reduce sphere-forming capability and induces cell death. (**a**) Cancer cells HeLa, HCT116, and 22Rυ1 were grown in low attachment plates, and E.C.M. detached cells were treated with different concentrations of GSK343 and Compound C for 2–8 days, and images were captured by Nikon inverted light microscope at ×40. Images were analyzed for size measurement using image J software (mean ± S.E.M., n = 8). A total number of 100 spheroids were quantified for size analysis; *P < 0.05. (**b**) Cancer cells HeLa, HCT116, and 22Rυ1 were grown in low attachment plates, and E.C.M. detached cells were treated with different concentrations of GSK343 and Compound C for 8 days, and apoptosis was quantified by using Annexin V/PI staining.

E.C.M. detachment of cancer cells has been shown to promote metabolic stress, which results in the activation of stress resistance kinase, i.e., AMPK, that supports cell survival. Recent finding reports that AMPK phosphorylates and thereby reduces the EZH2 methyltransferase activity. We also found a high expression of AMPK and EZH2 in matrix detached cancer cells (Supplementary Fig. S3). Therefore, we hypothesized that inhibiting the AMPK activity will promote the EZH2 activity and will further enhance the anti-proliferative impact of EZH2 inhibitor in E.C.M. detached cancer cells. As shown in Fig. 4, both AMPK kinase and EZH2 methyltransferase inhibitor, i.e., compound C and GSK 343 alone, significantly reduces the spheroid size in all the three cell lines. However, in the HeLa cell line, the combination of compound C and GSK 343 dramatically reduces the spheroid size from 4416 to 1672 mm^3^ on the 8th-day post-treatment, much stronger than the single inhibitor (Fig. 4a). Next, similar findings were observed in the HCT116 cell line where the combination of compound C and GSK 343 significantly reduces the spheroid size from 5120 to 648 mm^3^ on the 8th day when compared to a single treatment (Fig. 4a). Further, in 22Rυ1, a similar trend was observed, i.e., the combination of compound C and GSK 343 significantly reduces the spheroid size from 49,128 to 2988 mm^3^ on the 8th day when compared to single treatment (Fig. 4a).

Finally, in order to assess the impact of single vs. combination on E.C.M. detached cancer cells death, we performed apoptosis assay, and results showed that GSK 343 (Hela:27%; HCT116:16%; 22Rυ1: 13% approx.) and compound C (Hela:33%; HCT116:17%; 22Rυ1: 25% approx.) were to promote cell death in each cell line tested. However, the combination of both compound C and GSK 343 induces a profound, statistically significant, and better apoptosis-inducing impact (Hela:43%; HCT116:44%; 22Rυ1: 30% approx.) than the single treatment itself (Fig. 4b).

## Discussion

The major findings of the current work are (a) E.C.M. detached cancer cells induces EZH2 expression and activity. (b) Cell clustering after E.C.M. detachment is essential for the increased expression of EZH2. (c) Targeting EZH2 methyltransferase activity reduces the spheroid size. (d) Co-targeting heightened AMPK activity with EZH2 methyltransferase activity enhances the capability of GSK343 to repress spheroid size and to induce apoptosis.

E.C.M. detachment is a critical factor for the initiation of distant metastasis of primary tumors^14^. We have observed that during E.C.M. detachment cancer cells induce multiple histone methyltransferase in different cell lines. However, one methyltransferase that seems to be common was EZH2, which can induce transcriptional repression of key tumor suppressor genes in a wide variety of cancer types^23–27^. Our results are inconsistent with a recent finding where authors have shown induction of D.N.A. methylation in E.C.M. detached conditions using circulating tumor cells (C.T.C.s) of human origin as a model^28^. However, in our study, we mainly focused on histones and found that E.C.M. detachment induces repressive histone methylation in the spheroids by inducing EZH2 levels and activity. It would be intriguing to confirm the induction of repressive histone methylation and their possible functional impacts in the C.T.C.s in future work.

Targeting EZH2 activity, specifically in the E.C.M. detached conditions, showed a clear reduction in cell proliferation and viability. Next, by using apoptosis assay, we found strong induction of apoptosis during EZH2 activity inhibition, suggesting the critical role of the repressive EZH2 methylation in maintaining both proliferation and viability of cancer cells in E.C.M. detached conditions. In the past, some studies have linked the role of EZH2 with cancer cell metastasis and showed that targeting EZH2 reduces the metastatic burden^29,30^. However, we strongly believe that our data holds novelty as to the best of our knowledge no previous studies have shown high histone methylation and EZH2 induction in E.C.M. detached cancer cells. A clear outcome of the proliferation and viability assays was that the impact of EZH2 inhibition on E.C.M. detached cancer cells was independent of cell and cancer types, further suggesting that at least this might be a common mechanism for a variety of different cancer cells of different origins. This claim needs further experimentation validation.

In a recent report, AMPK mediated phosphorylation of EZH2 methyltransferase resulted in the loss of its enzymatic activity^26,31,32^. Further, AMPK induction is now well-established during E.C.M. detachment of cancer cells. Therefore, in our study, we presumed that inhibition of AMPK would result in higher enzymatic activity of EZH2 methyltransferase and higher sensitivity towards GSK343 inhibitor. Results clearly supported our hypothesis, and we found synergy between the AMPK and EZH2 inhibitor in reducing the cell viability of E.C.M. detached cancer cells. This result clearly suggests tailoring new therapeutic regimes for targeting E.C.M. detached and C.T.C.s.

Overall, we showed that EZH2 induction is critical for the survival and sphere formation of E.C.M. detached cancer cells. It would be interesting to see in the future whether EZH2 induction plays a critical role in promoting therapy resistance in these E.C.M. detached cancer cells or C.T.C.s.

## Materials and methods

### Cell culture

Tumorigenic cell lines, namely HeLa (cervical carcinoma), HCT-116 (colon carcinoma), and 22Rυ1 (prostate carcinoma) are from ATCC (Manassas, VA, U.S.A.). A short tandem repeat D.N.A. profiles database was utilized by ATCC for their authentication and characterization. Cells were cultured as per the recommendations of ATCC by using appropriate cell culture medium, i.e., RPMI-1640 and DMEM (Life Technologies, Carlsbad, CA, U.S.A.).

### Matrix detachment model

We employed two strategies for the induction of E.C.M. detachment: (1) Culture of cells using ultra-low attachment plates for short (8 h) and long (24 h) periods. (2) Culture of cells using poly-HEMA coated plates for short (8 h) and long (24 h) periods. Briefly, plates were prepared by layering 10 mg/ml poly-HEMA overnight in a CO_2_ incubator followed by washing using culture grade PBS and dried overnight at 37 °C. The assessments of E.C.M. detachment were done in cell suspension culture. Cells were dislodged by simple agitation in the presence of trypsin, followed with washing with PBS, resuspended at 0.5 × 10^6^ cells/ml in serum-free culture media containing B.S.A., and finally were cultured in poly-HEMA-coated plates at 37 °C for various time points, which results in the formation of spheroids. The spheroids were treated with either vehicle control or with different concentrations of GSK343 (Abcam, Cambridge, MA USA), Compound C (Abcam, Cambridge, MA USA) alone, or in combination for 2–8 days. The images were captured by using Nikon inverted light microscope. Images were analyzed for size measurement using image J software (https://imagej.net/Invasion_assay).

### Real-time qPCR analysis for mRNA expression

Briefly, R.N.A. was extracted from the E.C.M. attached, detached, treated, and non-treated with epigenetic inhibitor samples with the use of the RNeasy kit (Qiagen). Extracted R.N.A. was reverse-transcribed into cDNA with the use of iScript Reverse transcription supermix kit (Biorad), and 100 ng from this cDNA was amplified (done in triplicate) with the use of gene-specific primers as listed in Supplementary Table S1. The Threshold cycle (C_*T*_) values extracted from the instrument’s software were utilized in calculating the ΔC_*T*_ i.e. (C_*T*_ mRNA—C.T. housekeeping gene) and ΔΔC_*T*_ i.e. (ΔC_*T*_ experimental group − ΔC_*T*_ control group). The fold change was then quantified using the formula 2^−ΔΔCT^.

### Protein extraction and western blot analysis

Various cancer cells were cultured in a T_75_ flask (1 × 10^6^/flask). After 24 h of matrix detachment, the media of cells was aspirated, cells were washed with cold PBS (pH 7.4), and pelleted in 15 ml falcon tubes. An ice-cold lysis buffer was added to the pellet. The composition of lysis buffer was 50 mM Tris–HCl, 150 mM NaCl, 1 mM ethylene glycol-bis(aminoethyl ether)-tetraacetic acid, 1 mM ethylenediaminetetraacetic acid, 20 mM NaF, 100 mM Na_3_VO_4_, 0.5% NP-40, 1% Triton X-100, 1 mM phenylmethylsulfonyl fluoride, pH 7.4 with freshly added protease inhibitor cocktail (Protease Inhibitor Cocktail Set III, Calbiochem, La Jolla, CA). Then cells were passed through the needle of the syringe to break up the cell aggregates. The lysate was cleared by centrifugation at 14000*g* for 30 min at 4 °C, and the supernatant (whole-cell lysate) was used or immediately stored at − 80 °C. For western blotting, 10% polyacrylamide gels were used to resolve 15 μg of protein, transferred onto a nitrocellulose membrane, probed with appropriate monoclonal primary antibodies, and detected by chemiluminescence autoradiography after incubation with specific secondary antibodies.

### Histone extraction

The extraction of histone from both groups was achieved with the use of a commercial kit Epigentek Inc., U.S.A. (EpiQuik™ Total Histone Extraction Kit cat no: OP-0006-100). Briefly, 2–3 × 10^7^ million cells were utilized for histone isolation, and their protein contents were quantified using the standard B.S.A. method. The isolated histones were stored at − 80 °C until use.

### H3K27 methyltransferase activity assay

The enzyme activity of H3K27 specific methyltransferase was measured using a commercial kit (Abcam, H3K27 methylation colorimetric assay kit, catalog # ab113463). This assay kit can be used to measure H3K27 mono/di/tri subtypes activity or inhibition in cellular nuclear extracts. Briefly, protein samples in equal amounts were loaded for each independent experiment with an overall protein concentration ranged from 100 to 300 ng. The H3K27 modifications were quantified following the manufacturer’s instructions, with consideration for the amount of protein. For each modification, values were expressed as a percentage over untreated control.

### Analysis of cell death

Cells treated or non-treated during E.C.M. detached conditions were seeded in culture plates and were treated with different concentrations of AMPK (compound C), EZH2 (GSK343) inhibitor, or both for different time intervals. Apoptosis assay was performed by using Annexin V/PI solution (B.D. Biosciences), and the results were expressed as the percentage of apoptotic cells.

### Statistical analysis

Data were reported as mean ± S.D. Data analyses by two-tailed and unpaired t-test were done using GraphPad Prism (version 5; GraphPad Software). *p values < 0.05 were taken as being significant.

## Supplementary Information

Supplementary Information.

## Data Availability

All data generated or analysed during this study are included in this published article (and its Supplementary Information files).
