# Supplementary material for: Molecular profiling of epigenetic landscape of cancer cells during extracellular matrix detachment
Source: Sci Rep. 2021 Feb 2;11:2784. doi: 10.1038/s41598-021-82431-w (PMC7854657; doi:10.1038/s41598-021-82431-w)
Supplement: Supplementary file 1 — Supplementary Information. [file 41598_2021_82431_MOESM1_ESM.docx]

**Molecular profiling of epigenetic landscape of cancer cells during extracellular matrix detachment**

Mohammad Imran Khan, Mazin A. Zamzami, Aftab Ahmad and Hani Choudhry


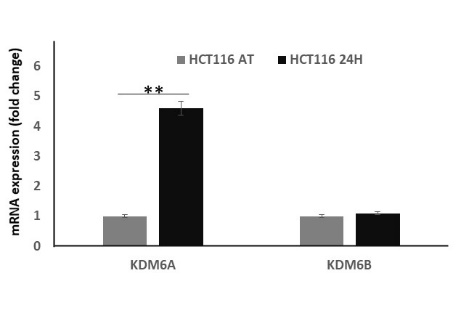

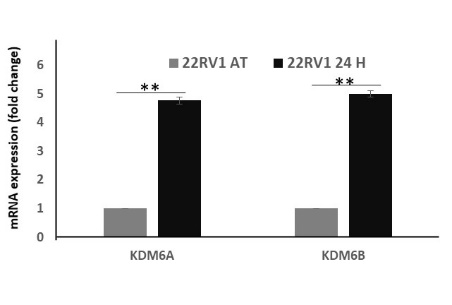
Supplementary Fig. S1


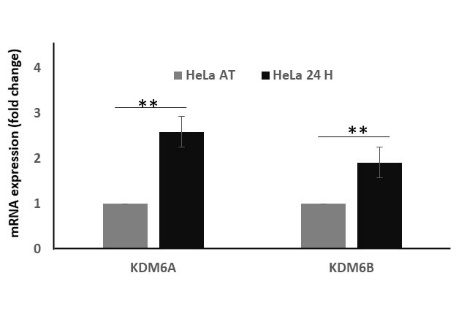


Impact of ECM detachment on H3K27 demthylases expression All three endogenous cancer cells were grown in an ultra-low attachment plate for 24-hour. After completion of time, RNA was isolated and quantitative-PCR was performed for targeted genes, values were normalized with housekeeping gene RPLP0. Error bars illustrate mean ± SEM.

Supplementary Fig. S2


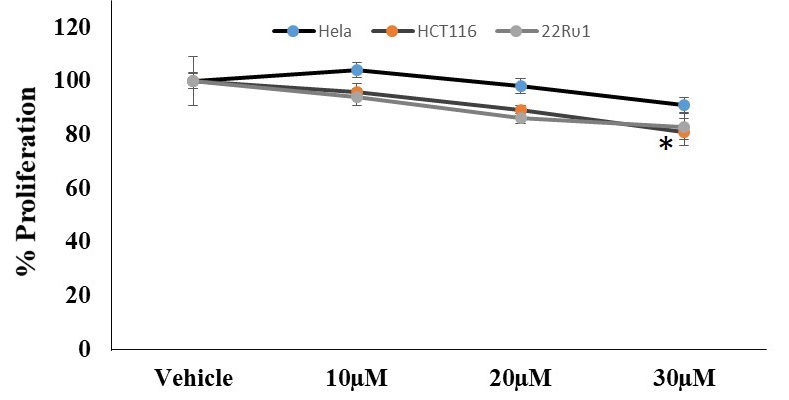


EZH2 methyltransferase activity inhibition by GSK343 shows weak cell viability inhibition in the attached cells. Different cancer cell lines namely 22Rυ1, HCT116, and HeLa have been treated with GSK343 and EZH2 specific inhibitor at different doses, and cell viability assay was performed at 48 hours; *P<0.05.

Supplementary fig. S3

1.
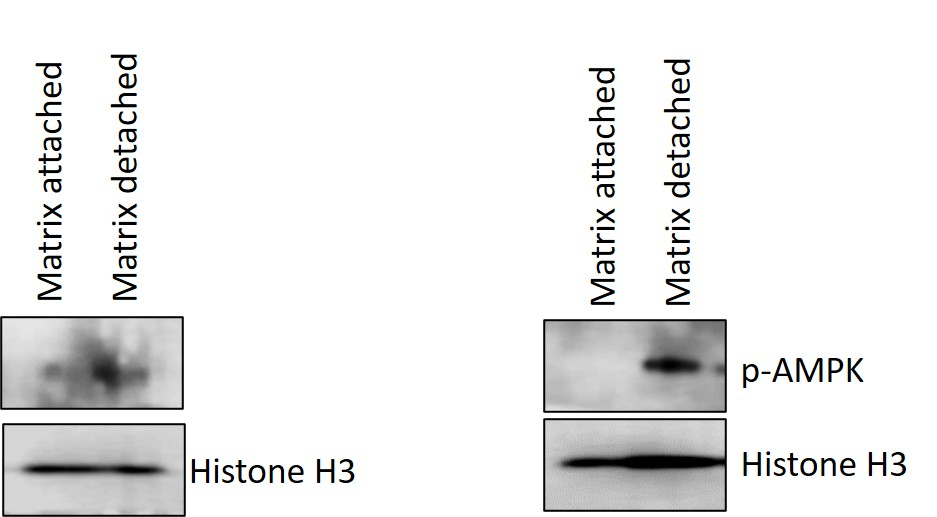
 (b)

Expression of EZH2 and p-AMPK in matrix attached and detached conditions in 22Rυ1 cells. Histone H3 was used as a loading control.


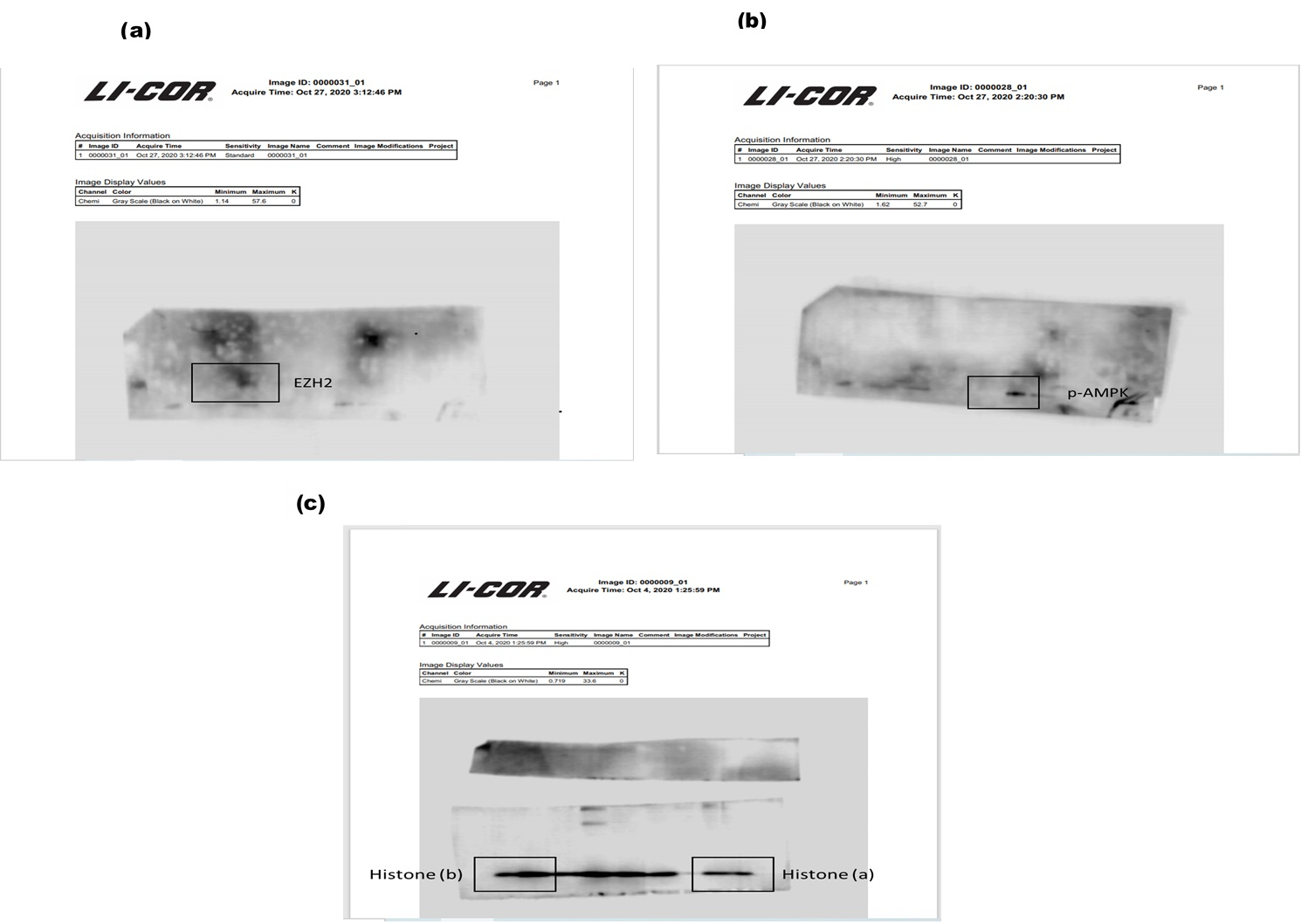
'

Full-length blots of Supplementary Fig S2. (**a**) The full-length blot of EZH2 in supplementary figure S2a. (**b**) The full-length blot of p-AMPK in supplementary figure S2b. (**c**)The full-length blot of histone loading control in supplementary figure S2

Supplementary Table S1

List of primer sequences used in the current study.

| **Gene name** | **Forward primer** | **Reverse primer** |
| --- | --- | --- |
| hARID1A | CGATGGGACCACGACAGC | GGCCCTATTCCAGGCTCC |
| hCARM1 | GCAACAGCGTCCTCATCCA | GCAGGTTTTCAGGATGTTGTAGAA |
| hDOT1L | GAGTGGAGGGAGCGAATCG | GATCCACCTCAGGACCAAAGG |
| hEHMT1 | GGAGGAACTGCCGAAATCG | GTCCCGCGTCCGGTAGA |
| hEHMT2 | TGATGTGAGAGAGGATGATTCTTACC | GCATCTATGCAGTACACCTCTCCAT |
| hEZH2 | GCTTTTCTGTAGGCGATGTTTTAAA | CCGCTTATAAGTGTTGGGTGTTG |
